# Dual-Gated Graph Convolutional Recurrent Unit with Integrated Graph Learning (DG3L): A Novel Recurrent Network Architecture with Dynamic Graph Learning for Spatio-Temporal Predictions

**DOI:** 10.3390/e27020099

**Published:** 2025-01-21

**Authors:** Yuxuan Wang, Zhouyuan Zhang, Shu Pi, Haishan Zhang, Jiatian Pi

**Affiliations:** National Center for Applied Mathematics in Chongqing, Chongqing Normal University, Chongqing 401331, China; wangyuxuan@stu.cqnu.edu.cn (Y.W.); 19913295129@163.com (Z.Z.); 2023110516043@stu.cqnu.edu.cn (S.P.); zhanghaishan0077@gmail.com (H.Z.)

**Keywords:** graph learning, spatio-temporal prediction, Graph Convolutional Network, Gated Recurrent Unit, attention mechanism

## Abstract

Spatio-temporal prediction is crucial in intelligent transportation systems (ITS) to enhance operational efficiency and safety. Although Transformer-based models have significantly advanced spatio-temporal prediction performance, recent research underscores the importance of learning dynamic spatio-temporal dependencies for these tasks. This paper introduces the Dual-Gated Graph Convolutional Recurrent Unit with Integrated Graph Learning (DG3L), a framework specifically designed to address the complex demands of spatio-temporal prediction. The DG3L model includes a memory-based graph learning module capable of generating dynamic graphs to accurately reflect ongoing changes in spatio-temporal dependencies. By integrating the strengths of Transformer and Graph Convolutional Recurrent Unit (GCRU) technologies within its Dual-Gated Graph Convolutional Recurrent Unit architecture, DG3L provides a mechanism for fusing Transformer features with contextual features from recurrent units. In practical applications, DG3L acts as an advanced representation learning module, delivering highly accurate context features for complex downstream tasks in ITS.

## 1. Introduction

Traffic flow prediction is a crucial component of modern urban management and Intelligent Transportation Systems (ITSs). It involves forecasting the movement of vehicles or pedestrians through a network of roads or routes over time. This process is shaped by both spatial and temporal dimensions; ‘spatial’ refers to various locations within the network such as intersections and road segments, while ‘temporal’ pertains to time intervals, often measured in minutes or hours. The data used for this prediction typically come in a time series format, where each point detailing traffic volume or density at a specific time and place provides essential insights. These data are meticulously collected through sensors, cameras, or GPS devices, offering a comprehensive, multidimensional view of urban traffic patterns. Such detailed data collection is fundamental as it sets the groundwork for effective traffic flow prediction [1], which is a pivotal task for enhancing road management, travel planning, and vehicle navigation. Accurate traffic prediction can improve system efficiency, reduce congestion, save energy, and support sustainable urban development [2]. However, traffic flow data often exhibit complex spatio-temporal heterogeneity, influenced by factors like weather, accidents, and road structure changes. This complexity makes traffic prediction a challenging task. Traditional statistical models often fail to capture the complex spatio-temporal dependencies in traffic flow, resulting in less accurate predictions.

In recent years, with the continuous development of big data technology and deep learning models, data-driven spatio-temporal forecasting methods have made significant progress. Initially, most models evolved primarily in two directions: methods based on Recurrent Neural Networks (RNNs) and those based on Convolutional Neural Networks (CNNs). Although RNN-based methods capture spatio-temporal dependencies by filtering inputs through Graph Convolutional Networks (GCNs) and passing the hidden states to recurrent units, they exhibit low efficiency when handling long sequences and are prone to gradient explosion when combined with GCN [3]. CNN-based methods, on the other hand, improve computational efficiency by combining graph convolution with standard one-dimensional convolution [2]. However, they still require stacking multiple layers or using global pooling to expand the model’s receptive field. As these methods have developed, research in the spatio-temporal forecasting field has gradually shifted toward Transformer-based models [4], which demonstrate significant advantages in capturing long-term dependencies. Unlike RNN models, which are more suitable for short-sequence forecasting, Transformer models utilize the Self-Attention Mechanism to overcome the limitations of RNN in long-term dependency modeling, thereby showing exceptional performance in long-term forecasting tasks. This self-attention mechanism enables Transformer models to capture complex, long-term spatio-temporal dependencies more efficiently, bringing a new breakthrough to spatio-temporal forecasting [5].

**Figure 1:**
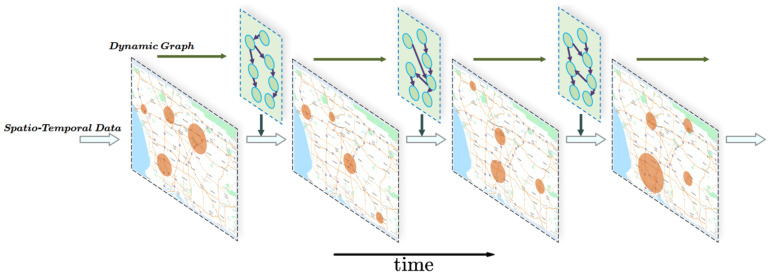
A typical framework for spatio-temporal forecasting using temporal dynamic graphs. Each time step introduces unique spatial dependencies, enhancing the representation of dynamic states. The dynamic graph learning module significantly reduces the main network’s pressure to accurately capture spatio-temporal variations, yet demands higher capabilities from the graph learning module.

Most traditional spatio-temporal forecasting models are designed based on static graphs. However, in many real-world applications, the graph evolves over time, known as a dynamic graph. As nodes, attributes, and edges change over time, the complexity and challenges of spatio-temporal forecasting tasks also increase. To address this, researchers have proposed several methods to handle dynamic graphs and integrate them with spatio-temporal forecasting. For example, a time graph embedding method based on random walks automatically samples subgraphs with connections within a specific time range, effectively capturing spatio-temporal dependencies [6]. Another method, DyANE [7], transforms the time graph into a static graph representation (hyper-adjacency representation), preserving time path information for subsequent embedding learning. Additionally, point process models have been applied to time graph representation learning, capturing node changes in dynamic graphs by modeling discrete time event sequences. DyRep [8] uses point processes to generate dynamic node embeddings and estimates the likelihood of edge occurrence at specific time stamps based on these embeddings. These dynamic graph learning methods demonstrate how to effectively capture and represent spatio-temporal dependencies in time-varying graphs, advancing spatio-temporal forecasting models to better adapt to more complex dynamic environments, thus improving both prediction accuracy and efficiency. Recent advancements include GW-Net [9], AdapGL [10], and STGM [11], which introduced an adaptive graph learning method using two learnable embedding matrices to dynamically generate graph structures based on spatio-temporal data. In the area of temporal dynamic graph generation, as shown in Figure 1, despite the innovations and certain achievements of DGCRN [12], challenges still arise from the inherent contradictions between the dynamic spatial dependencies of spatio-temporal data and the increased difficulty in model convergence due to the deepening complexity of graph learning models.

Although the aforementioned learning models generally perform well in spatio-temporal forecasting, they need to resolve the contradiction between representing complex spatio-temporal dependencies and achieving convergence stability in graph learning. Additionally, through multiple experiments, we believe that leveraging the short-term forecasting strengths of RNN structures combined with the long-term forecasting capabilities of Transformers is key to enhancing spatio-temporal prediction accuracy. To address these challenges, this paper introduces a new framework of Dual-Gated Graph Convolutional Recurrent Unit with Integrated Graph Learning. Our framework aims to learn the dynamic graph under low-rank conditions from the complex representations derived from the Transformer structure, while an improved GRU structure is used to integrate these complex representations to enhance contextual features, thereby capturing complex dependencies within spatio-temporal data. The specific contributions are as follows:

- We provide a framework that effectively combines the Transformer and GRU structures, while also outputting the spatio-temporal dependencies’ feature matrix and spatio-temporal features as results.
- Novel Dual-Gated Graph Convolutional Recurrent Unit (DG-GCRU): We design a new Graph Convolutional Recurrent Unit that integrates long-term information, short-term information, and adaptive embeddings for gated selection.
- New Memory Mechanism for Dynamic Graph Generation: We introduce a memory mechanism to generate a learnable dynamic graph adjacency matrix to optimize the representation learning of the DG-GCRU.
- We conduct multi-step and single-step traffic flow forecasting experiments on six real-world public datasets. The results demonstrate that our model achieves excellent performance on these datasets.

## 2. Related Work

**Traffic Forecasting.** As a typical task in multivariate time series forecasting, traffic prediction has long attracted extensive attention from researchers [1]. Early studies primarily relied on statistical models, such as the Autoregressive (AR) model, Vector Autoregression (VAR) [13], and the Autoregressive Integrated Moving Average (ARIMA) model [14]. These methods, which model historical data based on linear assumptions, achieved some success in the early stages. However, as the complexity of traffic data increased, these traditional methods struggled to effectively capture the nonlinear characteristics and spatio-temporal dependencies in traffic flow. To address this limitation, deep learning models have gradually gained attention, particularly long short-term memory (LSTM) [15] and Gated Recurrent Units (GRUs) [16], which effectively capture both short- and long-term dependencies through gating mechanisms and are widely used in traffic flow forecasting. Subsequently, the emergence of temporal convolutional networks, such as Graph WaveNet, further enhanced the ability to handle long-sequence dependencies [9]. Similarly, the ASTGCN [17] introduces a spatial–temporal attention mechanism to dynamically capture both spatial and temporal correlations of traffic data, significantly enhancing the forecasting accuracy. Following this, MAEGCLSTM [18] adds a Memory Attention module that captures global spatial dependencies and integrates a novel simplified GCLSTM with an encoder–decoder architecture, considerably improving multi-step traffic forecasting. In recent years, transformer models, owing to their superior performance in long-sequence modeling via the Self-Attention Mechanism, have driven advancements in traffic prediction technology [4]. Transformer models can flexibly model long-distance dependencies while adapting to complex spatio-temporal features. Consequently, various Transformer-based models have emerged to address the more complex spatio-temporal dependencies in traffic flow forecasting.

**Dynamic Graph Learning.** In traffic flow forecasting, early methods often relied on the natural topology of road networks or static graph structures based on certain predefined metrics [2,19]. However, these static graph structures struggle to adapt to the dynamic changes in traffic flow over time. To address this issue, GW-Net [9] introduced an adaptive graph learning method, using two learnable embedding matrices to dynamically generate the graph structure. This enables the model to adjust node relationships based on real-time traffic data, thus better capturing spatio-temporal dependencies. The adaptive graph concept in GW-Net has provided significant inspiration for subsequent studies. Building on this, AGCRN [20] introduced node-specific convolution filters, allowing each node’s characteristics to be better represented during the convolution process. CCRNN [21] further enhanced adaptability by learning multiple adaptive graphs through multi-layer graph convolution, capturing spatio-temporal dependencies at different levels. Additionally, StemGNN [22] combined the Self-Attention Mechanism to dynamically learn latent graph structures from input data, offering more flexible spatio-temporal dependency modeling capabilities. Expanding on dynamic graph structures, the DST-Trans [23] model utilizes a spatio-temporal transformer combined with a gated temporal convolutional network and Graph Convolutional Networks to effectively capture the dynamic spatio-temporal complexity of traffic flow, further enhancing the adaptability and predictive performance of traffic forecasting systems. Recent advancements include MTGNN [24], which utilizes mix-hop propagation layers and dilated inception layers to effectively capture both spatial and temporal dependencies in multivariate time series data. Similarly, D2STGNN [25] introduces a decoupled spatial–temporal framework that models diffusion and inherent signals within traffic data separately, enhancing the prediction of traffic flow dynamics. The introduction of DGCRN [12] and its hyper-networks for dynamic adjacency matrix generation marks a significant advancement in this field, further enhancing the dynamic nature and predictive performance of the models. Similarly, the integration of reinforcement learning in the Dynamic Graph Convolutional Network by Peng et al. [26] for long-term traffic flow prediction represents another innovative step towards addressing data deficiencies and improving spatio-temporal predictions. These methods showcase the substantial potential of dynamic graph learning in traffic flow forecasting, enabling models to flexibly adjust graph structures to accommodate continuously changing traffic data. Compared to early methods that rely on static topologies, adaptive graph structures significantly improve models’ adaptability to complex traffic environments, laying a solid foundation for high-precision traffic forecasting. These advancements also provide critical insights for future dynamic graph modeling, such as STGM [11], emphasizing that dynamically updating node relationships is key to enhancing spatio-temporal forecasting model performance.

**Gated Recurrent Unit.** In traffic forecasting, the Gated Recurrent Unit (GRU) is an efficient Recurrent Neural Network that regulates information flow through update and reset gates, effectively capturing both short-term and long-term temporal dependencies [27]. GRU is computationally efficient with a simple structure, making it widely used for tasks like traffic flow forecasting and travel time estimation. However, GRU primarily focuses on temporal dependencies and has limited ability to model spatial relationships within traffic networks, making it inadequate for handling dynamic spatial variations in complex traffic networks [28]. To improve spatio-temporal modeling, researchers have explored combining GRUs with Graph Convolutional Networks (GCNs). DCRNN [19] is a classic model in this area, which combines diffusion convolution with GRUs to capture diffusion dynamics in non-Euclidean spaces, better modeling the spatio-temporal propagation relationships among neighboring nodes. The GRU component handles temporal dependencies, significantly improving prediction performance in complex traffic networks. Additionally, Saravanan et al. proposed an improved deep hybrid model (CNN-rGRU) [29], combining convolutional layers with GRU for residual learning, which adaptively learns the spatio-temporal features of traffic congestion. Zhao et al. introduced a Temporal Graph Convolutional Network (T-GCN) [30], where GCN is used to learn complex spatial topologies and GRU captures the dynamic changes in traffic flow, making it suitable for city-road-network-based traffic forecasting. Building on this, MegaCRN [31] introduced a meta-learning framework that dynamically generates graph convolution weight matrices and combines them with recurrent networks to handle temporal dependencies, enhancing the model’s ability to personalize its predictions. MegaCRN adaptively adjusts graph convolution weights to reflect the characteristics of different traffic nodes, improving its ability to capture spatial dynamics.

**Transformer.** In traffic flow forecasting, the Transformer model has gradually become an important tool for handling spatio-temporal data due to its strong parallel processing and sequence modeling capabilities [4]. Traffic data often exhibit significant dynamism and complex spatial dependencies, which traditional statistical models, such as ARIMA and VAR, struggle to capture effectively. In contrast, the Transformer model leverages the self-attention mechanism to globally model dependencies in long time sequences while integrating multiple spatio-temporal dimensions, making it applicable to tasks like traffic flow, travel time prediction, and urban congestion pattern analysis. For example, Traffic Transformer introduces seven different temporal encoding mechanisms to handle continuity and periodicity in time series data [5]. These encodings help the model more accurately capture daily and weekly repeating patterns and, combined with Graph Convolutional Networks (GCN), model spatial dependencies within traffic networks. The attention mechanism can also identify the importance of data features, helping the model to understand the impact of sudden events, such as traffic accidents or holidays, on traffic flow and thereby improve prediction accuracy. GMAN [32] incorporates spatio-temporal embeddings for both input and output sequences and lies in the introduction of the Graph Multi-Attention Network (GMAN), which utilizes an encoder–decoder architecture with multiple spatio-temporal attention blocks to effectively model the impact of spatio-temporal factors on traffic conditions. STAEformer [33] offers a new approach based on spatio-temporal adaptive embedding. It uses an adaptive embedding layer to dynamically adjust embedding weights according to the spatio-temporal characteristics of traffic data, better capturing the dynamic relationships among nodes in the traffic network [31]. Unlike Traffic Transformer, which relies on fixed temporal encoding, STAEformer employs an adaptive mechanism to learn spatio-temporal pattern changes in real time within the traffic network. This dynamic adjustment allows STAEformer to perform more stably when processing complex traffic data, especially in large-scale long-term forecasting tasks, by reducing error accumulation in multi-step forecasting and improving prediction outcomes.

## 3. Problem Definition

The primary objective of traffic flow prediction is to forecast the flow within a traffic system over future time periods based on historical observation data. To ensure broad applicability, we define the problem as a multi-step prediction task as follows:

(1) $$\left[X_{t-(\alpha -1)},\cdots ,X_{t};G\right]\overset{\;f(\cdot )\;}{\underset{\theta }{\to }}\left[X_{t+1},\cdots ,X_{t+\beta }\right]$$

Let $X_{i}\in R^{N\times C}$, where *N* represents the number of spatial units (e.g., nodes, regions, roads), and *C* denotes the number of information channels. Additionally, G which is provided before the prediction task begins, represents the connectivity between these *N* spatial nodes. Given the observations from the previous α time steps $\left[X_{t-(\alpha -1)},\cdots ,X_{t}\right]$, our goal is to infer the traffic flow for the next β time steps $\left[X_{t+1},\cdots ,X_{t+\beta }\right]$ by training a predictive model *f* with parameters θ.

## 4. Methodology

**Figure 2:**
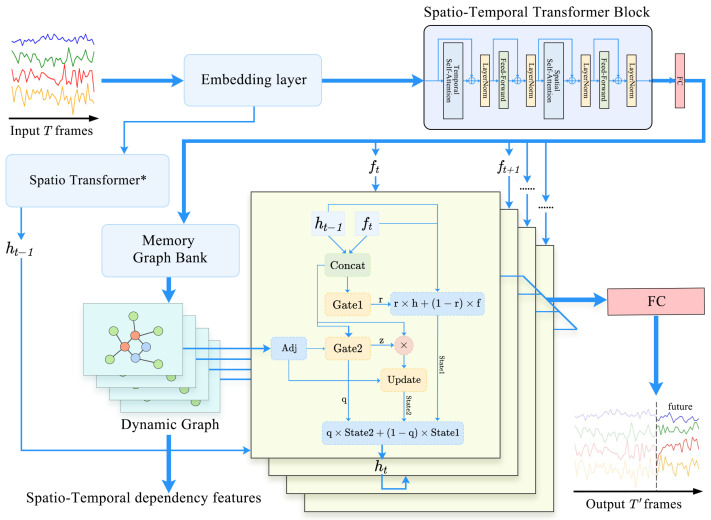
The framework of DG3L: For a spatio-temporal data input, the process begins with an Embedding module that concatenates time and adaptive embedding vectors. This is followed by a spatio-temporal transformer that outputs hidden vectors matching the output sequence length. On one side, it queries a Memory Graph Bank to generate graph sequences, and on the other, it feeds into the DG-GCRU module for representation blending. Finally, the DG-GCRU performs graph convolution operations within the recurrent network based on the generated graph sequences. The output sequence from DG-GCRU is then processed through a fully connected layer to produce the desired predictive output.

Our DG3L framework, as illustrated in Figure 2, primarily consists of a graph learning module and a Dual-Gated Graph Convolutional Recurrent Unit (DG-GCRU). The graph learning module is employed to extract spatio-temporal dependency information, while the Dual-Gated GCRU is utilized for sequence prediction.

### 4.1. Transformer-Based Memory Graph Bank for Graph Learning

Our Graph Learning module is composed of two parts: the Spatio-Temporal Transformer and the Memory Graph Bank.

**Embedding Layer.** In common spatio-temporal prediction tasks, spatio-temporal embeddings [32] related to time and space are crucial. Leveraging the adaptive embedding module from the STAEformer [33], known for its effectiveness, we incorporate this module along with time embedding across all our embedding layers. This integration is crucial for preserving the integrity of the raw data. We employ a linear layer to derive the feature embedding $E_{f}\in R^{T\times N\times d_{f}}$, a spatio-temporal adaptive embedding $E_{a}\in R^{T\times N\times d_{a}}$, and the periodicity embedding $E_{p}^{t}\in R^{T\times d_{p}}$:

(2) $$E_{f}=\mathrm{Linear}\left(X_{t-T+1:t}\right)$$

(3) $$\Gamma_{0}=E_{f}∥E_{p}∥E_{a}$$

Here, $d_{f}$ represents the dimension of the feature embedding and Linear(·) denotes a linear transformation layer that operates on the last dimension of the input, mapping $R^{d_{in}}\to R^{d_{f}}$. By concatenating these embeddings, we obtain a hidden spatio-temporal representation $\Gamma_{0}\in R^{T\times N\times d_{h}}$, where the hidden dimension $d_{h}$ equals $d_{f}+d_{p}+d_{a}$.

**Temporal Transformer.** In the Temporal Transformer, we transpose the spatio-temporal representation Γ to $\Gamma \in R^{N\times T\times d_{h}}$. The transformation is governed by:

(4) $${\mathrm{SelfAttention}}_{T}\left(\Gamma \right)=A\;V\left(\Gamma \right)=\mathrm{Softmax}\left(\frac{Q\left(\Gamma \right)K{\left(\Gamma \right)}^{⊤}}{\sqrt{d_{h}}}\right)V\left(\Gamma \right)$$

(5) $$\left\{\begin{array}{cc} \Gamma_{l}^{*} & =\mathrm{LayerNorm}({\mathrm{SelfAttention}}_{T}\left(\Gamma_{l}\right)+\Gamma_{l})\\ \Gamma_{l+1} & =\mathrm{LayerNorm}(\mathrm{FeedForward}\left(\Gamma_{l}^{*}\right)+\Gamma_{l}^{*}) \end{array}\right.$$

where Q(Γ), K(Γ), V(Γ) are linear layers, and the matrix multiplication $Q\left(\Gamma \right)K{\left(\Gamma \right)}^{⊤}$ acts on the last dimension, enabling $A\in R^{N\times T\times T}$ to capture the temporal relations across different spatial nodes.

**Spatio Transformer.** This component mirrors the structure of the Temporal Transformer, with the input spatio-temporal representation Γ transposed to $\Gamma \in R^{T\times N\times d_{h}}$. Consequently, $A\in R^{T\times N\times N}$ captures the spatial relations across different time steps.

**Spatio-Temporal Transformer.** We achieve our framework by sequentially connecting the Temporal and Spatio Transformers:

(6) $$F=\mathrm{SpatioTransformer}(\mathrm{TemporalTransformer}\left(\Gamma_{0}\right))$$

**Memory Graph Bank Section.** Inspired by the innovative concept of the Meta-Node Bank (MegaCRNN [31]), which effectively generates a context vector, we propose utilizing time-series data within memory network structures to construct dynamic time-series graphs. To this end, we have developed a Dynamic Graph Memory Bank, denoted as $B\in R^{\phi \times d}$, where ϕ represents the number of memory nodes and *d* denotes the dimension of each memory node. The primary functionalities of this memory bank are outlined as follows:

(7) $$\left\{\begin{array}{cc} Q_{1} & =F\cdot W_{1}+b_{1}\\ Q_{2} & =F\cdot W_{2}+b_{2}\\ E_{1} & =\sum_{j=1}^{\phi }\left(\frac{\exp(Q_{1}\cdot B^{⊤}\left[j\right])}{\sum_{k=1}^{\phi }\exp\left(Q_{1}\cdot B^{⊤}\left[k\right]\right)}\right)B\left[j\right]\\ E_{2} & =\sum_{j=1}^{\phi }\left(\frac{\exp(Q_{2}\cdot B^{⊤}\left[j\right])}{\sum_{k=1}^{\phi }\exp\left(Q_{2}\cdot B^{⊤}\left[k\right]\right)}\right)B\left[j\right] \end{array}\right.$$

Here, $Q_{1}$ and $Q_{2}$ are query vectors derived from the hidden spatio-temporal representation *F* through two distinct linear layers. Both $E_{1}$ and $E_{2}$, which reside in $R^{T\times N\times \phi }$, serve as memory vectors extracted from the memory bank *B* via two sophisticated attention mechanisms. These vectors facilitate the formation of a dynamic graph, $DynAdj\in R^{T\times N\times N}$ defined by:

(8) $$DynAdj=\mathrm{softmax}(\mathrm{relu}\left(E_{1}\cdot E_{2}^{⊤}\right))$$

This dynamic graph is generated from the memory vectors through a softmax function, which reflects an entropy-driven approach to understanding graph structures, capturing the spatio-temporal relationships within the dataset.

**Contrastive Loss and Consistency Loss.** To further refine our model’s ability to discern and differentiate between various temporal patterns in the data, we have adopted the InfoNCE loss [34], inspired by recent advancements in graph generation methodologies [35]. The InfoNCE loss is particularly effective in enhancing the discriminative capabilities of models by maximizing mutual information between closely related samples while minimizing it among less related ones. The loss is defined as follows:

(9) $${\mathrm{Loss}}_{\mathrm{InfoNCE}}=\sum_{t\in T_{0}}\left(-\log\frac{\exp(Q_{1,t}\cdot B\left[p\right]/\tau )}{\sum_{n=1}^{N}\exp\left(Q_{1,t}\cdot B\left[n\right]/\tau \right)}-\log\frac{\exp(Q_{2,t}\cdot B\left[p\right]/\tau )}{\sum_{n=1}^{N}\exp\left(Q_{2,t}\cdot B\left[n\right]/\tau \right)}\right)$$

Here, $Q_{1,t}$ and $Q_{2,t}$ are the query vectors derived from the hidden spatio-temporal representations at time step *t*. B[p] represents the positive memory item within the memory bank, which is semantically similar to the query vectors at the corresponding time steps and is selected based on the highest similarity scores to enhance learning from relevant contrasts. Conversely, B[n] denotes the set of negative samples, which are chosen based on lower similarity scores to provide diverse comparisons, thereby improving the discriminative training of the model. τ is the temperature parameter that scales the dot product within the softmax function in the InfoNCE loss, controlling the sharpness of the distribution. $T_{0}$ specifically refers to the set of the first time steps across all output sequences, crucial for establishing the initial context for the model’s learning process. The InfoNCE loss discriminates node information in the graph by maximizing mutual information between similar samples and minimizing it among dissimilar ones. This concept extends Cross-Entropy Loss to an unsupervised context, focusing on distinguishing complex patterns rather than predicting exact categories. To further understand this idea, consider the following reformulation of the Cross-Entropy Loss:

(10) $${\mathrm{Loss}}_{\mathrm{Cross}\text{-}\mathrm{Entropy}}=-\log\left(\frac{\exp(z_{\mathrm{true}})}{\sum_{j=1}^{K}\exp\left(z_{j}\right)}\right)=-\log\left(\frac{\exp(\mathrm{sim}\left(\phi_{i},\phi_{i}^{+}\right))}{\sum_{j=1}^{K}\exp\left(\mathrm{sim}\left(\phi_{i},\phi_{j}^{-}\right)\right)}\right)$$

In the unsupervised task, $\mathrm{sim}(\phi_{i},\phi_{i}^{+})$ is denoted as $z_{\mathrm{true}}$, representing the logit of the true class. The parameter *K* refers to the number of classes in the Cross-Entropy context, which corresponds to the number of samples in a batch for InfoNCE, indicating the range of summation. Additionally, $\mathrm{sim}(\phi_{i},\phi_{j}^{-})$ quantifies the dissimilarity between the target sample $\phi_{i}$ and a negative sample $\phi_{j}^{-}$, serving a role similar to the logits of non-target classes in Cross-Entropy. InfoNCE loss includes a temperature parameter τ to control the sharpness of the similarity distribution, thereby influencing the model’s sensitivity to differences in similarity.

Similar to the approach used in [36], we also apply a consistency loss to ensure node stability across a temporal graph:

(11) $${\mathrm{Loss}}_{\mathrm{consistency}}=\sum_{t\in T_{0}}\left(\left|\right|Q_{1,t}-{B\left[p\right]||}^{2}+\left|\right|Q_{2,t}-B\left[p\right]{\left|\right|}^{2}\right)$$

This loss minimizes the variance in embeddings over time, promoting temporal coherence and ensuring that B[p] serves as a stable reference point within the memory bank. To effectively train our model, we combine multiple loss components into a unified training objective:

(12) $${\mathrm{Loss}}_{\mathrm{train}}={\mathrm{Loss}}_{\mathrm{prediction}}+\alpha \cdot {\mathrm{Loss}}_{\mathrm{InfoNCE}}+\beta \cdot {\mathrm{Loss}}_{\mathrm{consistency}}$$

where ${\mathrm{Loss}}_{\mathrm{prediction}}$ is the primary function for accuracy, and α and β are coefficients that regulate the impact of the InfoNCE and consistency losses on the training process.

In these regularization terms, our decision to compute the contrastive and consistency losses only at the first time step $T_{0}$ of the output sequence is based on our understanding of recurrent networks. This approach allows the network to establish a strong initial context, which is crucial for accurately modeling the subsequent dynamics of the sequence.

### 4.2. Dual-Gated Graph Convolutional Recurrent Unit

To enhance the model’s performance, we directly use the output $H_{0}$ of a single layer of the Spatio Transformer as the input of decoder, rather than concatenating it with the output of the GCRU-Encoder. This decision is based on our belief that when the input time series is lengthy, the Transformer’s self-attention mechanism can effectively process all time steps in parallel. Referring to (4) and (5), the initial transformation is given by

(13) $$H_{0}={\mathrm{SpatioTransformer}}^{*}\left(\Gamma_{0}^{\prime }\right)$$

Here, $\Gamma_{0}^{\prime }$ is a reshaped version of $\Gamma_{0}$, where the temporal dimension T and the feature dimension $d_{h}$ are merged. This transformation is mathematically represented as $\Gamma_{0}^{\prime }\in R^{N\times (T*d_{h})}$. The ${\mathrm{SpatioTransformer}}^{*}$ module is a SpatioTransformer with a fully connected layer (FC) preceding it, refining feature extraction. This transformation can be formally represented as a function $f:R^{N\times (T*d_{h})}\to R^{N\times d_{h}}$.

Since the Spatio-Temporal Transformer has already learned spatial–temporal representations for better capturing spatio-temporal relationships, we aim for the GCRU module to leverage these representations fully. This allows the model to harness the strengths of the Transformer in long-sequence prediction tasks and the GCRU in short-sequence prediction tasks. Therefore, we enhance the GCRU module and introduce the Dual-Gated Graph Convolutional Recurrent Unit (DG-GCRU).

**GCN (Chebyshev GCN).** Before detailing the DG-GCRU, it is crucial to understand the underlying graph convolution operation. The graph convolution function [37], denoted as GCN, is defined as follows:

(14) $$GCN\left(V,Adj\right)=\sigma \left(\sum_{k=0}^{K}Adj^{k}\;V\;W_{k}\right)$$

Here, *V* denotes the input features, and Adj represents a snapshot of the dynamic adjacency matrix, known as DynAdj, at a specific time point. This adjacency matrix, $Adj\in R^{N\times N}$, encapsulates the spatial relationships and dependencies among features at that time instant. The graph convolution operation employs Chebyshev polynomials up to order *K* to approximate the parameters $W_{k}$ of the kernel. An activation function σ is subsequently applied to the output feature matrix, facilitating the effective processing of connected features through the graph structure.

**Fusion Gate.** In traditional GRU modules, the interaction between the candidate states and intermediate states is essential for effective sequence modeling. To improve feature integration from the Transformer, we introduce a novel gating mechanism, termed Fusion Gate. This gate effectively balances the contextual information from previous time steps in the GRU with the current time step information processed by the Transformer’s attention mechanisms, as described below:

(15) $$\left\{\begin{array}{cc} r_{t} & =\mathrm{sigmoid}(W_{r}\cdot \left[X_{t},F_{t},H_{t-1}\right]+b_{r})\\ M_{t} & =r_{t}⊙H_{t-1}+\left(1-r_{t}\right)⊙F_{t} \end{array}\right.$$

Here, $r_{t}$ is the fusion gates, $W_{r}$ is the weight matrix applied to the concatenated inputs and the previous hidden state to compute the reset gate, $X_{t}$ is the input at time step *t*, $F_{t}$ is the feature input, $H_{t-1}$ is the hidden state from the previous time step, and $b_{r}$ is the bias term for the fusion gate. $M_{t}$ is the new intermediate state, ⊙ denotes element-wise multiplication and $\left(1-r_{t}\right)$ is the complement of the fusion gate.

**GCRU (Graph Convolutional Recurrent Unit).** Although the Transformer can output the feature input $F_{t}$ in parallel with the input $X_{t}$, we naturally believe that the representation learned by $F_{t}$ is not entirely accurate, and it should also be controlled by a reset gate. Therefore, we design

(16) $$\left[z_{1t},z_{2t},q_{t}\right]=\mathrm{sigmoid}\left(GCN\left(\left[X_{t},F_{t},H_{t-1}\right],DynAdj_{t}\right)+b_{z}\right)$$

where $z_{1t}$ and $z_{2t}$ are reset gates, $q_{t}$ is the update gate, $X_{t}$ is the input at time step *t*, and $b_{z}$ is the bias term for the update gate. $DynAdj_{t}\in R^{N\times N}$, which is a dynamic adjacency matrix at time step *t*. The sigmoid function ensures that the values of $z_{1t}$, $z_{2t}$, and $q_{t}$ are between 0 and 1, controlling the flow of information.

(17) $$C_{t}=\tanh\left(GCN\left(\left[X_{t},z_{1t}⊙H_{t-1},z_{2t}⊙F_{t}\right],DynAdj_{t}\right)+b_{C}\right)$$

$C_{t}$ is the candidate state, and $b_{C}$ is the bias term for the candidate state. The integration of the candidate state $C_{t}$ with the update mechanism through $q_{t}$ allows for a dynamic adjustment of the hidden state, reflecting both new inputs and historical data.

(18) $$\left\{\begin{array}{cc} H_{t} & =q_{t}⊙M_{t}+\left(1-q_{t}\right)⊙C_{t}\\ \hat{Y} & =\mathrm{FC}(H) \end{array}\right.$$

Here, $H_{t}$ represents the hidden state at time step *t*, and $\left(1-q_{t}\right)$ acts as the complement of the update gate. This equation integrates the intermediate state $M_{t}$ and the candidate state $C_{t}$ based on the update gate $q_{t}$, determining the final hidden state. The collection of hidden states across all time steps is denoted by $H=[H_{t-(\alpha -1)},\ldots ,H_{t}]$, where *H* is a tensor in $R^{T\times N\times d_{h}}$, encapsulating the results from all time steps. $\hat{Y}$ denotes the output predictions. The function FC, a fully connected layer, performs a linear transformation mapping $R^{d_{h}}\to R$, projecting high-dimensional hidden states to scalar outputs for sequence modeling predictions.

## 5. Experiment

In the experimental section of this study, all model data were obtained from the BasicTS library [38]. BasicTS provides a unified framework and standardized data processing, allowing for more objective and accurate comparisons between different models in multivariate time series forecasting tasks. In our experimental design, we relied on BasicTS’s implementations of each model to ensure that all models were evaluated under the same conditions, guaranteeing fairness and consistency in the results.

### 5.1. Experimental Setup

**Datasets.** Our method was comprehensively validated on six benchmark datasets for traffic flow forecasting: PEMS03, PEMS04, PEMS07, PEMS08, METR-LA, and PEMS-BAY. The first two datasets were proposed by DCRNN [19] and contain traffic speed data collected from 207 sensors in Los Angeles and 325 sensors in the Bay Area, respectively. The latter four datasets were introduced by STSGCN [39]. All datasets are sampled at 5-min intervals, resulting in 12 frames per hour. Detailed information on the datasets is provided in Table 1.

**Settings.** Our model implementation utilizes PyTorch and operates on a Linux server with a GeForce RTX 4080 GPU. The dataset partitioning is as follows: METR-LA and PEMS-BAY datasets are split into training, validation, and test sets at a 7:1:2 ratio, while the PEMS03, PEMS04, PEMS07, and PEMS08 datasets are divided at a 6:2:2 ratio. The dimension $d_{h}$, comprising components $d_{f}$, $d_{p}$, and $d_{a}$, maintains a ratio of 2:1:1. For the PEMS03, PEMS04, PEMS07, and PEMS08 datasets, $d_{h}$ is configured to 32, whereas for PEMS-BAY and METR-LA, $d_{h}$ is set to 28. Both spatial and temporal Transformers have 1 layer, with an attention head count of 4. Additionally, the number of memory nodes for all datasets is set to 20, and the memory dimension is configured to 32. The input and prediction durations are each set to 1 h. During training, the batch size is generally 64, though it is reduced to 16 for the PEMS07 dataset due to the higher node count. We utilize the Adam optimizer with an initial learning rate of 0.008, which gradually decreases during training. Training is conducted over 100 epochs, with early stopping applied if the validation error stabilizes over 50 consecutive steps to prevent overfitting. To ensure reliability, each experimental result is averaged across three independent training runs.

**Metrics.** We use two widely adopted metrics for the traffic forecasting task, namely MAE and RMSE. We evaluate the average performance of all 12 forecasting time steps across the PEMS03, PEMS04, PEMS07, PEMS08, METR-LA, PEMS-BAY datasets. For the METR-LA and PEMS-BAY datasets, we compare the performance at the 3rd, 6th, and 12th time steps (15, 30, and 60 min) to provide a more detailed evaluation.

### 5.2. Performance Evaluation

Table 2 presents a comprehensive comparison of the performance of our model, DG3L, against several state-of-the-art baseline models across six traffic flow forecasting datasets: PEMS03, PEMS04, PEMS07, PEMS08, METR-LA, and PEMS-BAY. The evaluation metrics include MAE (Mean Absolute Error), RMSE (Root Mean Square Error), and MAPE (Mean Absolute Percentage Error). The results illustrate that our method consistently achieve superior performance on most datasets and metrics, particularly excelling in minimizing MAE and RMSE values. In the more challenging PEMS03 dataset, DG3L outperforms other models, with an MAE of 14.51 and an RMSE of 25.69, showcasing its effectiveness even in larger and more complex datasets. Similarly, in the PEMS04 dataset, DG3L continues to lead, with an MAE of 18.22 and an RMSE of 29.82. It is noteworthy that STWave and STAEformer also show strong performances across various horizons, indicating their potential in handling multi-step predictions. The PEMS07 dataset, known for its high number of nodes, sees our DG3L model maintaining a competitive edge with an MAE of 19.59 and an RMSE of 33.07. This dataset highlights the strengths of the D2STGNN and STAEformer models, which achieve the best and second-best performances, respectively, suggesting their suitability for complex network structures. Finally, on the PEMS08 dataset, DG3L achieves the best MAE of 13.72 and an excellent RMSE of 23.09, closely followed by STAEformer with an MAE of 13.43 and an RMSE of 23.31. These results underscore the adaptability and efficiency of our DG3L model across different traffic conditions and forecasting horizons. On the METR-LA dataset, our model, DG3L, demonstrates robust performance with an MAE of 2.90 and an RMSE of 5.98, which are competitive figures close to the best results shown by D2STGNN with an MAE of 2.87 and an RMSE of 5.90. This indicates a strong predictive accuracy in urban traffic contexts. For the PEMS-BAY dataset, DG3L also shows excellent results, achieving the lowest errors with an MAE of 1.54 and an RMSE of 3.57, closely followed by D2STGNN, which recorded an MAE of 1.52 and an RMSE of 3.53. Overall, our DG3L model not only consistently achieves top-tier performance but also demonstrates significant improvements over other models like DCRNN and STGCN, which struggle with larger datasets. The comparative analysis with models such as GWNet, STNorm, and particularly D2STGNN and STAEformer, highlights the nuanced capabilities of these models in specific scenarios, reinforcing the importance of tailored approaches in traffic flow forecasting.

### 5.3. Ablation Study

To evaluate the effectiveness of each component in our model, we conducted an ablation study using three variants of the model, as shown in Table 3.

- **w/o Dual-Gate.** This variant removes the Fusion Gate and the reset gate $z_{2}$ of the *F* vector from the Spatio-Temporal Transformer.
- **w/o MemoryGraph-Bank.** This variant removes the memory-based dynamic graph generation mechanism.
- **w/o SpatioTransformer-Encoder.** This variant replaces the Spatio Transformer (one layer) with a GCRU as the encoder.

### 5.4. Hyperparameter Sensitivity

**Figure 3:**
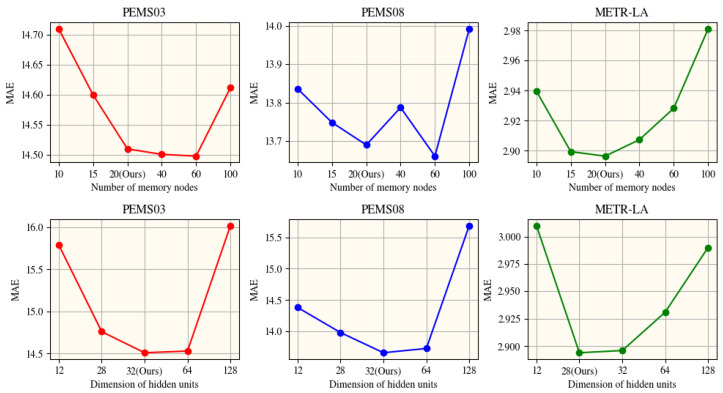
The variation in Mean Absolute Error (MAE) across the PEMS03, PEMS08, and METR-LA datasets as influenced by the number of memory nodes and the dimension of hidden units.

Our studies indicate that while the DG3L model presents some susceptibility to variations in hyperparameters like $d_{h}$ and memory nodes, these do not radically alter overall model performance, except beyond certain thresholds. These insights stress the need for nuanced hyperparameter tuning to optimize performance without overscaling the model’s complexity. We conducted an investigation into the hyperparameter sensitivity of the DG3L model, as depicted in Figure 3. Our focus was on understanding the effects of the dimension of hidden units ($d_{h}$) and the number of memory nodes.

Through this study, we discovered that setting the hidden dimension $d_{h}$ to approximately 30 achieves an effective equilibrium between processing capacity and model complexity. We directly define $d_{h}$ by the proportional relationship $d_{f}:d_{p}:d_{a}$ = 2:1:1. This edit retains the essential information and smoothly transitions into the dimension ratio while properly referencing the related equation as requested. The impact of the number of memory nodes varied notably across the PEMS03, PEMS08, and METR-LA datasets. A moderate increase typically improves the model performance; however, exceeding a certain threshold leads to performance degradation due to escalating complexity. Each dataset demonstrated unique responses, identifying distinct optimal memory configurations beyond which further increases diminished returns on performance. These findings underscore the importance of meticulously adjusting hyperparameters like the number of memory nodes in accordance with the specific characteristics and computational constraints of the dataset. Such customization is vital to harnessing the full potential of the DG3L model in diverse data environments.

### 5.5. Efficiency Study

**Figure 4:**
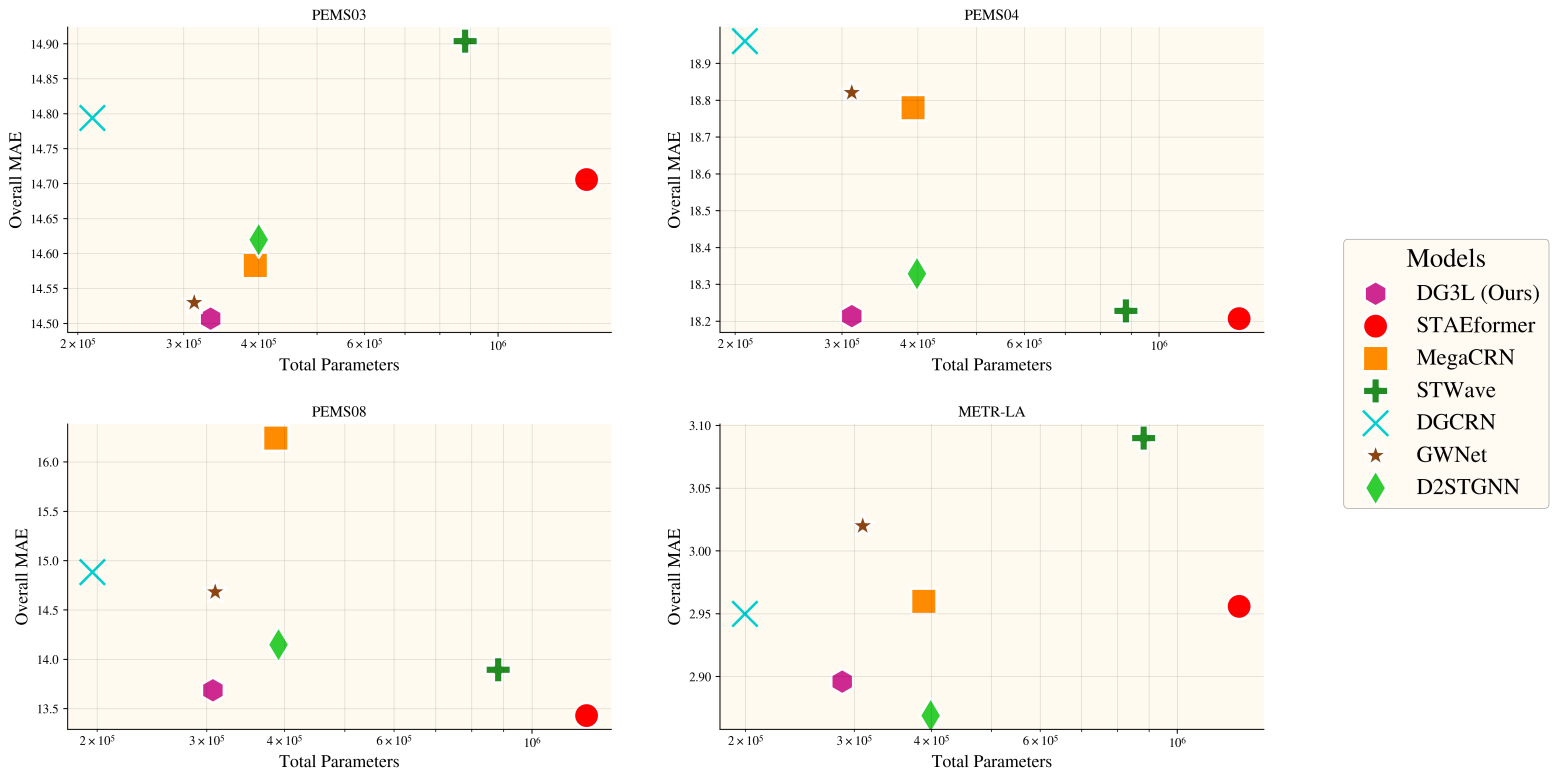
This scatter plot illustrates the comparison of Mean Absolute Error (MAE) across various models, indicated by different markers, in relation to the total number of parameters within each dataset.

In our efficiency study, we evaluated the DG3L model’s performance across four datasets, PEMS03, PEMS04, PEMS08, and METR-LA, comparing it with other state-of-the-art models. According to the results illustrated in Figure 4, for the PEMS03 dataset, DG3L, with 332,556 parameters, outperformed MegaCRN in terms of overall MAE and exhibited strong parameter efficiency. On the PEMS04 dataset, it maintained competitive accuracy with 311,285 parameters, showing a lower MAE than MegaCRN and demonstrating considerable efficiency. The PEMS08 dataset highlighted DG3L’s superior accuracy with 306,957 parameters, once again surpassing MegaCRN in overall performance. In the METR-LA dataset, while DG3L achieved excellent parameter efficiency with 286,733 parameters, D2STGNN managed a marginally better MAE. Throughout these analyses, DG3L consistently exhibits a favorable balance of parameter efficiency and competitive accuracy across diverse settings without compromising its performance.

## 6. Conclusions

In this paper, we introduced the DG3L framework, a sophisticated approach designed for predicting traffic flow within intelligent transportation systems. The DG3L framework incorporates a memory-based dynamic graph learning module coupled with a dual-gated mechanism. This configuration enables the effective capture of spatio-temporal characteristics of traffic flows, facilitating the generation of dynamic dependency matrices and spatio-temporal feature vectors. These elements are crucial for accurately modeling and predicting traffic flow patterns. Our experimental evaluations, as detailed in the performance comparison tables, affirm that DG3L consistently outperforms traditional methods, particularly in monitoring traffic at critical nodes and forecasting traffic flows. The framework’s flexibility in constructing dynamic spatio-temporal correlations is evident, with its memory mechanism playing a pivotal role in capturing long-term dependencies. This enhances both the prediction accuracy and the utility of contextual features, rendering DG3L highly suitable for real-time traffic forecasting and alert scenarios in intelligent transportation systems. However, it is important to acknowledge certain limitations. The primary datasets used in testing DG3L were existing spatio-temporal datasets, which may not encompass the full complexity of real-world spatio-temporal networks or unstructured data. To overcome these limitations and ensure broader applicability, further validation in more diverse and complex spatio-temporal scenarios is essential. As the field of dynamic graph learning progresses, there is potential to extend DG3L’s functionality by integrating newer datasets and models, thereby enhancing its adaptability to a wider array of spatio-temporal data challenges. We encourage researchers and practitioners interested in advancing traffic flow prediction models within intelligent transportation systems to utilize the DG3L framework by accessing the code https://github.com/cjwyx/DG3LFrame (accessed on 24 November 2024).

## Figures and Tables

**Table 1:** Summary of datasets.

Dataset	#Spatial Units	Time Interval	#Timesteps	Start Time	End Time
METR-LA	207	5 min	34,272	3/2012	6/2012
PEMS-BAY	325	5 min	52,116	1/2017	5/2017
PEMS03	358	5 min	26,209	5/2012	7/2012
PEMS04	307	5 min	16,992	1/2018	2/2018
PEMS07	883	5 min	28,224	5/2017	8/2017
PEMS08	170	5 min	17,856	7/2016	8/2016

**Table 2:** Comparison of various forecasting models using the metrics MAE, RMSE, and MAPE across multiple datasets and forecasting horizons, highlighting the top and second-best performances with bold/underlined and bold formatting, respectively.

Datasets	Methods	Overall			Horizon 3			Horizon 6			Horizon 12		
		MAE	RMSE	MAPE	MAE	RMSE	MAPE	MAE	RMSE	MAPE	MAE	RMSE	MAPE
**PEMS03**	DCRNN	15.476	27.579	15.80%	14.239	25.096	15.10%	15.486	27.799	15.77%	17.492	30.778	17.61%
	STGCN	15.947	27.324	15.58%	14.854	25.520	14.44%	15.884	27.379	15.46%	17.763	29.905	17.14%
	GWNet	**14.522**	**25.111**	15.36%	**13.379**	**23.107**	14.64%	**14.525**	**25.164**	15.30%	**16.273**	**27.805**	17.05%
	STNorm	15.341	25.909	**14.42%**	14.304	23.930	**13.61%**	15.573	26.362	**14.27%**	16.860	28.403	**16.01%**
	MTGNN	14.812	**25.074**	14.97%	13.777	23.629	14.28%	14.820	**25.161**	14.77%	16.485	**27.758**	16.53%
	STWave	14.904	26.122	15.41%	13.683	24.088	14.36%	14.786	25.929	15.14%	16.474	28.539	16.82%
	DGCRN	14.794	26.525	15.13%	13.695	24.475	14.68%	14.888	26.819	15.19%	16.539	29.262	16.15%
	MegaCRN	14.683	26.101	15.80%	13.572	24.346	15.16%	14.821	26.383	15.88%	16.467	28.733	17.37%
	D2STGNN	14.620	25.891	15.36%	13.501	23.843	14.46%	14.635	26.148	15.24%	16.364	28.507	16.80%
	STAEformer	14.706	26.046	15.39%	13.692	23.542	14.38%	14.972	26.133	15.35%	16.831	29.288	17.11%
	DG3L	**14.510**	25.689	**14.38%**	**13.201**	**23.533**	**13.48%**	**14.521**	25.623	**14.39%**	**16.324**	28.331	**16.06%**
**PEMS04**	DCRNN	19.652	31.174	13.73%	18.457	29.405	12.91%	19.663	31.192	13.73%	21.571	33.792	15.22%
	STGCN	19.729	31.445	13.66%	18.788	29.881	13.27%	19.702	31.419	13.78%	21.369	33.849	14.39%
	GWNet	18.821	30.157	13.18%	17.879	28.777	12.49%	18.828	30.289	13.06%	20.337	32.212	14.61%
	STNorm	19.196	32.195	12.96%	18.424	30.521	12.56%	19.303	32.564	13.08%	20.485	34.378	13.63%
	MTGNN	19.196	31.463	13.41%	18.325	29.864	12.78%	19.294	31.679	13.55%	20.605	33.758	14.25%
	STWave	18.228	**29.975**	**12.15%**	**17.469**	**28.703**	**11.67%**	**18.179**	**29.963**	**12.06%**	19.608	**31.763**	**12.93%**
	DGCRN	18.961	30.899	12.94%	17.962	29.103	12.33%	19.002	30.984	12.98%	20.597	33.453	14.00%
	MegaCRN	18.780	30.275	13.13%	17.743	28.676	12.54%	18.825	30.339	13.21%	20.446	32.651	14.23%
	D2STGNN	18.329	30.043	12.52%	17.565	**28.626**	12.13%	18.367	30.301	12.64%	19.600	**32.078**	13.44%
	STAEformer	**18.207**	30.391	**12.39**%	17.475	28.998	11.95%	**18.232**	30.512	12.40%	**19.320**	32.279	**13.14%**
	DG3L	**18.216**	**29.817**	12.41%	**17.473**	28.832	**11.83%**	18.241	**30.235**	**12.39%**	**19.520**	32.593	13.44%
**PEMS07**	DCRNN	21.433	34.910	9.02%	19.613	31.601	8.29%	21.410	34.898	8.97%	24.431	39.562	10.32%
	STGCN	22.066	35.669	9.41%	20.549	32.800	8.84%	22.001	35.539	9.36%	24.688	39.942	10.44%
	GWNet	20.356	33.346	8.67%	18.855	30.814	8.08%	20.374	33.409	8.61%	22.777	36.963	9.77%
	STNorm	20.644	34.996	8.72%	19.240	31.757	8.15%	20.783	35.224	8.80%	22.805	38.953	9.70%
	MTGNN	21.343	34.326	9.44%	19.444	31.254	8.34%	21.258	34.224	9.23%	24.509	38.767	11.43%
	STWave	19.919	33.876	8.40%	18.585	30.757	7.84%	19.917	33.198	8.41%	21.905	36.392	9.40%
	DGCRN	23.328	36.491	10.51%	19.548	31.275	8.48%	22.202	34.866	9.97%	30.626	45.671	14.28%
	MegaCRN	22.288	34.954	10.45%	20.279	31.789	9.65%	22.241	34.903	10.51%	25.452	39.402	11.88%
	D2STGNN	**19.566**	**32.631**	8.19%	18.164	**30.111**	7.68%	19.691	**32.678**	8.20%	**21.555**	**36.245**	**9.06%**
	STAEformer	**19.394**	**32.724**	**8.10%**	**18.091**	30.247	**7.57%**	**19.398**	**32.745**	**8.07%**	**21.446**	**36.197**	**8.99%**
	DG3L	19.593	33.068	**8.17%**	**18.077**	**30.161**	**7.59%**	**19.578**	33.021	**8.13%**	21.798	37.012	9.09%
**PEMS08**	DCRNN	15.199	24.199	10.23%	14.140	22.198	9.51%	15.217	24.264	10.19%	16.886	26.925	11.51%
	STGCN	16.171	25.392	10.47%	15.101	23.470	9.88%	16.066	25.338	10.44%	18.020	28.199	11.40%
	GWNet	14.684	23.610	9.74%	13.696	21.764	9.02%	14.675	23.597	9.77%	16.181	26.109	10.59%
	STNorm	15.413	24.912	9.84%	14.457	22.808	9.11%	15.484	25.049	9.96%	16.910	27.613	11.07%
	MTGNN	15.231	24.062	9.88%	14.256	22.277	9.10%	15.200	24.111	9.71%	16.831	26.577	11.25%
	STWave	13.896	24.175	9.14%	13.021	22.316	8.63%	13.804	24.246	9.09%	**15.021**	26.237	9.94%
	DGCRN	14.884	23.775	9.92%	13.693	21.695	8.96%	14.843	23.791	9.81%	16.811	26.645	11.45%
	MegaCRN	16.244	25.265	11.01%	14.641	22.738	9.93%	16.127	25.143	10.65%	18.910	29.011	12.32%
	D2STGNN	14.151	23.583	9.11%	13.206	21.539	8.50%	14.164	23.567	9.10%	15.498	26.103	10.05%
	STAEformer	**13.431**	**23.313**	**8.97%**	**12.545**	**21.429**	**8.41%**	**13.430**	**23.315**	**8.92%**	**14.787**	**25.828**	**9.74%**
	DG3L	**13.720**	**23.088**	**8.91%**	**12.742**	**21.229**	**8.38%**	**13.686**	**23.078**	**8.87%**	15.107	**25.632**	**9.92%**
**METR-LA**	DCRNN	3.039	6.248	8.34%	2.676	5.188	6.88%	3.076	6.291	8.43%	3.560	7.490	10.41%
	STGCN	3.093	6.268	8.35%	2.742	5.268	7.08%	3.133	6.321	8.48%	3.587	7.434	10.09%
	GWNet	3.031	6.121	8.14%	2.689	5.143	6.89%	3.072	6.176	8.28%	3.510	7.257	9.88%
	STNorm	3.144	6.475	8.77%	2.817	5.523	7.51%	3.204	6.590	9.00%	3.594	7.540	10.42%
	MTGNN	3.021	6.160	8.18%	2.685	5.175	6.88%	3.056	6.194	8.28%	3.492	7.294	10.00%
	STWave	3.102	6.465	8.79%	2.794	5.509	7.42%	3.144	6.527	8.86%	3.503	7.471	10.44%
	DGCRN	3.067	6.333	8.08%	2.678	5.173	6.75%	3.101	6.371	8.19%	3.606	7.636	9.90%
	MegaCRN	2.962	6.043	8.00%	2.611	4.996	6.68%	2.998	6.073	8.12%	3.461	7.247	9.84%
	D2STGNN	**2.869**	**5.895**	**7.83%**	**2.558**	**4.953**	**6.54%**	**2.904**	**5.938**	**7.92%**	**3.336**	**7.032**	**9.71%**
	STAEformer	2.956	5.999	7.93%	2.698	5.203	7.00%	2.993	6.072	8.19%	**3.341**	**7.022**	**9.68%**
	DG3L	**2.899**	**5.973**	**7.92%**	**2.554**	**4.971**	**6.51%**	**2.927**	**5.984**	**7.90%**	3.390	7.210	9.72%
**PEMS-BAY**	DCRNN	1.592	3.700	3.59%	1.312	2.765	2.73%	1.652	3.765	3.72%	1.970	4.615	4.71%
	STGCN	1.619	3.691	3.67%	1.351	2.829	2.87%	1.680	3.777	3.81%	1.982	4.548	4.70%
	GWNet	1.598	3.702	3.52%	1.306	2.753	**2.68%**	1.656	3.776	3.65%	1.992	4.613	4.60%
	STNorm	1.578	3.653	**3.49%**	1.329	2.826	2.76%	1.649	3.782	**3.64%**	1.913	4.442	4.45%
	MTGNN	1.596	3.665	3.51%	1.327	2.792	2.77%	1.654	3.747	3.65%	1.971	4.541	4.49%
	STWave	1.576	3.609	3.53%	1.333	2.840	2.73%	1.631	3.699	3.65%	1.916	4.361	4.48%
	DGCRN	1.565	3.619	3.54%	1.300	2.739	2.71%	1.621	3.694	3.67%	1.933	4.489	4.60%
	MegaCRN	1.558	3.635	3.53%	1.292	2.723	2.70%	1.616	3.715	3.66%	1.916	4.505	4.60%
	D2STGNN	**1.516**	**3.533**	**3.43%**	**1.259**	**2.660**	**2.63%**	**1.574**	**3.626**	**3.57%**	**1.863**	4.348	**4.42%**
	STAEformer	1.564	3.583	3.55%	1.325	2.794	2.82%	1.630	3.666	3.72%	1.878	**4.306**	**4.41%**
	DG3L	**1.539**	**3.571**	**3.49%**	**1.290**	**2.718**	**2.68%**	**1.601**	**3.652**	**3.64%**	**1.871**	**4.333**	**4.42%**

**Table 3:** Ablation Study on PEMS03, METR-LA, and PEMS-BAY with best performances in bold.

Datasets	PEMS03		METR-LA		PEMS-BAY	
Metric	MAE	RMSE	MAE	RMSE	MAE	RMSE
w/o Dual-Gate	14.647	26.329	2.947	6.094	1.562	3.607
w/o Memory Graph-Bank	15.492	27.936	3.117	6.466	1.580	3.683
w/o SpatioTransformer-Encoder	14.543	26.156	2.913	6.017	1.541	3.579
Ours	**14.510**	**25.689**	**2.899**	**5.973**	**1.539**	**3.571**

## Data Availability

The data presented in this study are available on request from the corresponding author.
